# The Burden of OASIS Increases along with Socioeconomic Position – Register-Based Analysis of 980,733 Births in Finland

**DOI:** 10.1371/journal.pone.0073515

**Published:** 2013-08-27

**Authors:** Sari Räisänen, Rufus Cartwright, Mika Gissler, Michael R. Kramer, Seppo Heinonen

**Affiliations:** 1 Department of Obstetrics and Gynaecology, Kuopio University Hospital, Kuopio, Finland; 2 Department of Epidemiology and Biostatistics, Imperial College London, London, United Kingdom; 3 Information Department, National Institute for Health and Welfare (THL), Helsinki, Finland; 4 Nordic School of Public Health, Gothenburg, Sweden; 5 Department of Epidemiology, Rollins School of Public Health, Emory University, Atlanta, Georgia, United States of America; 6 Department of Obstetrics and Gynaecology, Kuopio University Hospital, Kuopio, Finland; 7 Institute of Clinical Medicine, University of Eastern Finland, Kuopio, Finland; VU University Medical Center, The Netherlands

## Abstract

**Background:**

Obstetric anal sphincter injury (OASIS) has been identified as a major preventable risk factor for anal incontinence.

**Objective:**

Aim was to measure national variation in incidence of OASIS by socioeconomic status (SES).

**Methods:**

A retrospective population based case-control study using the data derived from the Finnish Medical Birth Register for the years 1991–2010. A total population of singleton vaginal births was reviewed. We calculated unadjusted incidences of OASIS stratified by SES and vaginal parity, and adjusted risks for OASIS in each social class, after controlling for parity, birthweight, mode of delivery, maternal age and maternal smoking. SES was recorded into five categories based on mother’s occupation at time of birth; upper white-collar workers such as physicians, lower white-collar workers such as nurses, blue-collar workers such as cleaners, others such as students, and cases with missing information.

**Results:**

Seven per thousand (6,404 of 980,733) singleton births were affected by OASIS. In nulliparae the incidence of OASIS was 18% higher (adjusted OR 1.18 95% CI 1.04−1.34) for upper white-collar workers and 12% higher (adjusted OR 1.12 95% CI 1.02−1.24) for lower white-collar workers compared with blue-collar workers. Among women in these higher SES groups, 40% of the excess OASIS risk was explained by age, non-smoking, birthweight and mode of delivery. Despite the large effect of SES on OASIS, inclusion of SES in multivariable models caused only small changes in estimated adjusted effects for other established risk factors.

**Conclusions:**

OASIS at the first vaginal delivery demonstrates a strong positive social gradient. Higher SES is associated with a number of risk factors for OASIS, including higher birthweight and non-smoking, but only 40% of the excess incidence is explained by these known risk factors. Further research should address other underlying causes including differences in lifestyle or environmental factors, and inequalities in healthcare provision.

## Introduction

Obstetric anal sphincter injury (OASIS) has been identified as a major preventable risk factor for anal incontinence (AI), with a majority of women with OASIS suffering with persistent AI symptoms [1]. The demographic and obstetric risk factors for OASIS are well-established, including first vaginal birth [2], [3], high birthweight [4], [5], prolonged active second stage of birth [3], vacuum and forceps deliveries [2], [5], and midline episiotomy [6]–[8]. We previously reported that smoking during pregnancy was associated with a decreased risk of OASIS, even after adjustment for birthweight [9]. In contrast to this negative association with OASIS, smoking has typically been identified as a risk factor for pelvic floor disorders in later life, including moderate associations with a variety of bladder storage symptoms [10], urinary incontinence (UI) [11], and faecal incontinence [12]. Importantly for the interpretation of the association of maternal smoking and OASIS, smoking during pregnancy is heavily underreported to maternity care providers,[13] and has become an important marker of socioeconomic deprivation [14]. Although estimates are heterogeneous, a number of large high quality studies have suggested that pelvic floor disorders may be more prevalent among women of higher socioeconomic status [15]–[17] and at least one single centre study has reported a similar positive socioeconomic gradient for OASIS [18].

The present study therefore aimed to measure the national variation in incidence of OASIS with socioeconomic status (SES) among the total population of singleton births in Finland (with around 5.5 million residents and mainly publicly funded health services) and to reassess the known risk factors for OASIS, including smoking, after adjustment for socioeconomic status.

## Methods

The data was gathered from the Finnish Medical Birth Register (MBR) that is currently maintained by National Institute for Health and Welfare (THL). The MBR includes information on maternal and neonatal birth characteristics and perinatal outcomes (all live births or stillbirths delivered after the 22nd gestational week or weighing 500 g or more) of all of the obstetric care units in Finland. For each infant, an electronic form, or rarely a paper form, has to be filled in by the hospital covering the first seven postnatal days. The validity of the data is guaranteed by active collaboration of delivery units and the register controller. All information submitted to the MBR is checked, and missing or inferred to be incorrect information are confirmed by contacting the treating hospitals before correction. The data is supplemented with data compiled by the Population Register Centre on live births and with data compiled by Statistics Finland on stillbirths and deaths during the first week of life. Some newborns (less than 0.1%) are missing in direct submissions to the MBR, but after additions from the Population Register Centre and Statistics Finland the coverage for birth events is 100%.

Information on OASIS has only been collected in the MBR since 2004, so for the years 1991−2003, the information about OASIS was taken from the Hospital Discharge Register (HDR), based on the International Classification of Diseases (ICD). Codes (ICD-9) were 6642B and 6643B for the years 1991−1995 and O70.2 (3^rd^ degree) and O70.3 (4^th^ degree) (ICD-10) for the years 1996−2003. The HDR established in 1969 contains information on all aspects of inpatient care and outpatient visits in Finnish hospitals thus we had also information concerning all aspects of care during pregnancy and birth such as medical interventions and surgical procedures. The MBR and the HDR were linked together using parturients’ encrypted unique personal identification numbers.

The degree of OASIS was classified according to standard definitions: a third degree rupture involves the external anal sphincter and a fourth degree rupture affects both the anal sphincter and the anorectal mucosa [19]. In all of the analyses, data on third and fourth degree OASIS were pooled. Gestational age was estimated based on data for the last menstrual period, unless there was a discrepancy of more than seven or 14 days at the first- or second-trimester ultrasonography measurements, respectively. Self-reported maternal smoking was recorded as non-smoking, smoking and gave up smoking during the first trimester of the pregnancy. Information on socioeconomic status (SES) was based on Finland's national Classification of Occupations that based on the international recommendations. SES was recorded into five categories based on mother’s occupation at time of birth; upper white-collar workers such as physicians, teachers and lawyers, lower white-collar workers such as nurses, secretaries and cashiers, blue-collar workers such as cleaners, cookers and dress-makers, and others as used previously [20]. These others mixed women from previous mentioned SES groups and included women whose SES could not be described such as entrepreneurs, housewives, students, retired, and unemployed as well as women with unclassifiable occupation. Information on SES was missing in 128,317 (13.1%), and these missing cases were therefore included in multivariate analyses as a separate category. The study period of 1991−2010 was divided into five time periods (1991−1995, 1996−2000, 2001−2005, 2006−2010) to examine the secular trends in OASIS incidence and risks.

Apart from maternal age, gestational age and birthweight all other variables were dichotomous or categorical in all the analyses. Mode of delivery was grouped as vaginal spontaneous, breech, forceps and vacuum assisted. Parity was dichotomized as either nulliparous (including women admitted for a first vaginal delivery after a prior cesarean section (CS)) or multiparous (second or subsequent vaginal delivery).

### Participants

The data included all singleton vaginal births (n = 980,733) for the years 1991–2010 in Finland.

### Ethics

The National Institute for Health and Welfare (THL) gave us the required authorization as required by national data protection legislation (Reference number 1749/5.05.00/2011). Studies utilizing anonymized register data do not require ethical committee approval in Finland according to the current data protection legislation. The register keeping organisation (THL) made the ethical evaluation of the study when they gave its permission to use the sentitive health register data in scientific research.

### Statistical methods

Statistical differences in frequencies (categorical and dichotomous variables) between women affected by OASIS and controls were evaluated by Chi Square test. The differences between continuous variables were evaluated by Mann Whitney U or Student’s t tests. Multivariable logistic regression analyses were used to model the risk factors of OASIS in comparison with controls separately for both parity groups. Odds ratios (OR) with 95% confidence intervals (CI) were calculated. Candidate independent variables were selected based on bivariate associations (p<0.05). Furthermore, to quantify contribution of risk factors for OASIS (age, smoking, birthweight and mode of delivery) to socioeconomic disparities in OASIS in nulliparous women including women with first vaginal delivery by using logistic regression. Each variable was added separately to model 2 (adjusted by SES and age), and the percentage reduction in the odds ratio was measured. The formula used was: (OR Model 2 – OR Model 3/4/5/6)/(OR Model 2 – 1) [21]. The data were analyzed using SPSS for Windows 19.0, Chicago, IL.

## Results

In total 0.7% (6,404 of 980,733) of singleton births were affected by OASIS between 1991 and 2010. Among nulliparous women (including women admitted for a first vaginal delivery after a prior CS), the incidence of OASIS was 1.2% (4,939 of 417,671) compared with 0.3% (1,465 of 563,062) among the multiparous women (Table 1).

**Table 1:** Delivery characteristics and interventions were compared between women delivered vaginally with and without OASIS within the groups of nulliparous women including women admitted for first vaginal delivery after a prior cesarean section for their first birth (n = 417,671) and multiparous women (n = 563,062) between 1991 and 2010 in Finland.

Delivery intervention/characteristic	Nulliparae and women with first vaginal delivery after a prior Cesarean section for their first birth			Multiparae		
% or Mean (SD)	With OASIS (1.2%, n = 4,939), % or mean (SD)	Without OASIS, % or mean (SD)	p value*	With OASIS (0.3%, n = 1,465), % or mean (SD)	Without OASIS, % or mean (SD)	p value*
Mean maternal age (SD) (year)	28.3(4.8)	27.0 (5.0)	≤0.001	30.7 (4.8)	30.6 (5.0)	0.51
Mean gestational age (SD) (wk)	40.3 (1.3)	39.9 (1.7)	≤0.001	40.2 (1.2)	39.9 (1.6)	≤0.001
Mean birthweight (SD) (g)	3683.7 (453.4)	3459.8 (504.3)	≤0.001	3866.8 (486.4)	3642.0 (516.2)	≤0.001
Mode of delivery			≤0.001			≤0.001
Vaginal delivery	0.9	99.1		0.2	99.8	
Breech	0.9	99.1		0.3	99.7	
Forceps	3.4	96.6		0.9	99.1	
Vacuum assistance	2.8	97.2		1.0	99.0	
Induction	1.1	98.9	≤0.001	0.3	99.7	0.15
Augmentation with oxytocin	1.2	98.8	0.001	0.3	99.7	0.003
Episiotomy	1.2	98.8	0.82	0.3	99.7	0.001
Epidural analgesia	1.3	98.7	≤0.001	0.3	99.7	≤0.001
Socioeconomic group			≤0.001			0.001
Upper white-collar worker	1.4	98.6		0.3	99.7	
Lower white-collar worker	1.1	98.9		0.3	99.7	
Blue-collar worker	0.9	99.1		0.2	99.8	
Other	1.2	98.8		0.2	99.8	
Missing	1.5	98.5		0.3	99.7	
Prior cesarean section	1.9	98.1	≤0.001	0.3	99.7	0.19
Smoking status			≤0.001			≤0.001
Non-smoking	1.3	98.7		0.3	99.7	
Quitted smoking	1.1	98.9		0.2	99.8	
Smoking	0.7	99.3		0.2	99.8	
Time periods			≤0.001			≤0.001
1991−1995	0.3	99.7		0.1	99.9	
1997−2000	0.6	99.4		0.1	99.9	
2001−2005	1.6	98.1		0.6	99.4	
2006−2010	2.3	97.7		0.3	99.6	

OASIS =  obstetric anal sphincter injuries, SD = standard deviation, (*Chi Square, Mann-Whitney U or Student’s t tests).

SES was strongly associated with incidence of OASIS. Among the nulliparous women, the incidence of OASIS varied from 1.4% in the highest SES category, down to 0.9% among women in the lowest category. There were strong associations between SES and all major risk factors for OASIS, with women in higher SES strata smoking less, having higher maternal age, higher birthweight, and more assisted operative deliveries (all p<0.001, Table 2). After adjustment for these potential mediators of the effect, nulliparous upper white collar workers and lower white-collar workers still had increased risk of OASIS compared to blue-collar workers (adjusted odds ratio (aOR) 1.18 95% CI 1.04−1.34 and aOR 1.12 95% CI 1.02−1.24 respectively, Table 3).

**Table 2:** Characteristics and background information were compared in nulliparous women including women with first vaginal delivery after a prior cesarean section between SES groups in 1991−2010 in Finland.

Characteristic	Upper white-collar worker	Lower white-collar worker	Blue-collar worker	Others a	Missing	p value*
OASIS %	1.4	1.1	0.9	1.2	1.5	≤0.001
Mean maternal age (SD) (year)	30.1 (4.0)	27.8 (4.5)	25.8 (4.7)	26.0 (5.3)	26.4 (5.4)	≤0.001
Mean gestational age (SD) (wk)	39.9 (1.7)	39.9 (1.7)	39.9 (1.8)	39.9 (1.7)	39.9 (1.9)	≤0.001
Mean birthweight (SD) (g)	3480.3 (491.8)	3470.6 (500.2)	3451.0 (515.5)	3465.1 (498.7)	3441.0 (516.7)	≤0.001
Mode of delivery %						≤0.001
Vaginal spontaneous	61.4	63.0	65.4	68.0	65.2	
Breech	0.4	0.4	0.4	0.5	0.6	
Forceps	0.2	0.3	0.3	0.2	0.2	
Vacuum	12.6	11.7	11.0	11.0	12.2	
Cesarean section^ b^	25.4	24.6	22.9	20.3	21.8	
Epidural analgesia %	55.1	53.3	51.9	58.0	58.3	≤0.001
Smoking status %						≤0.001
Non-smoking	95.0	85.4	71.5	83.7	79.6	
Quitted smoking	1.4	3.4	5.1	3.7	4.7	
Smoking	3.6	11.3	23.4	12.6	15.7	

a The ‘other’ group included entrepreneurs, students, retired, unemployed, housewives and unclassifiable cases,^b^ Additional information, *Chi Square or Analysis of Variance (ANOVA) tests.

**Table 3:** Adjusted odds ratio (OR) of OASIS in nulliparous women including women admitted for first vaginal delivery after a prior cesarean section for their first birth (n = 408,272) and multiparous women (n = 547,257) women with singleton births between 1991 and 2010 in Finland (Logistic regression).

Delivery intervention/characteristic	Nulliparae and women with first vaginal delivery adjusted OR (95% CI)	Multiparous women adjusted OR (95% CI)
Maternal age (year)		
≤19	1	
20−29 (≥29 ref multip)	1.56 (1.31−1.85)	1
30−39	1.81 (1.52−2.16)	0.98 (0.88−1.10)
≥40	1.34 (0.99−1.83)	0.93 (0.72−1.22)
Gestational age (wk)		
<37	1	1
37−39+6	1.43 (1.13−1.81)	1.15 (0.71−1.84)
40−41+6	1.49 (1.18−1.89)	1.28 (0.79−2.06)
>42	1.65 (1.27−2.15)	1.35 (0.76−2.38)
Birthweight (g)		
<3000	1	1
3000−3499	1.65 (1.45−1.88)	1.50 (1.09−2.07)
3500−3999	2.44 (2.14−2.78)	2.28 (1.67−3.12)
≥4000	3.94 (3.43−4.53)	3.64 (2.65−5.01)
Mode of delivery		
Vaginal delivery	1	1
Breech	1.04 (0.68−1.61)	0.77 (0.32−1.86)
Forceps	4.44 (3.18−6.21)	4.97 (1.21−20.34)
Vacuum	2.54 (2.38−2.71)	2.79 (2.26−3.44)
Prior cesarean section	1.38 (1.26−1.50)	1.01 (0.81−1.27)
Induction	0.98 (0.91−1.06)	0.96 (0.83−1.10)
Augmentation with oxytocin	1.02 (0.95−1.08)	0.95 (0.85−1.07)
Episiotomy	0.98 (0.92−1.05)	1.70 (1.49−1.94)
Epidural analgesia	0.87 (0.81−0.92)	0.88 (0.77−1.01)
Socioeconomic group		
Upper white-collar worker	1.18 (1.04−1.34)	0.95 (0.75−1.19)
Lower white-collar worker	1.12 (1.02−1.24)	1.07 (0.91−1.25)
Blue-collar worker	1	1
Other a	1.13 (1.02−1.25)	0.88 (0.74−1.04)
Missing	1.17 (1.05−1.30)	1.06 (0.88−1.29)
Smoking status		
Non-smoking	1	1
Quitted smoking	0.74 (0.64−0.87)	0.47 (0.29−0.77)
Smoking	0.80 (0.72−0.89)	0.85 (0.70−1.03)
Time periods		
1991−1995	1	1
1996−2000	1.80 (1.57−2.06)	1.85 (1.41−2.43)
2001−2005	5.13 (4.55−5.78)	12.51 (9.98−15.67)
2006−2010	7.14 (6.35−8.03)	7.67 (6.06−9.71)

a The ‘other’ group included entrepreneurs, students, retired, unemployed, housewives and unclassifiable cases.

Despite the large effect of SES, and its association with other risk factors for OASIS, inclusion of SES as a covariate caused only small changes in estimated effects for the other risk factors (Table 2). Among nulliparous women we observed an independent effect of both persistent smoking during pregnancy (aOR 0.74 95% CI 0.64−0.87, Table 3), and giving up smoking during pregnancy (aOR 0.80, 95% CI 0.72−0.89, Table 3). We observed similar associations with smoking status among multiparous women, although narrowly missing significance criteria for persistent smoking.

To separately quantify the contribution of maternal age, smoking, birthweight and mode of the delivery to SES disparities among nulliparous women we constructed nested logistic regression models, and calculated percentage reductions in each OR, as shown in Table 4. Model 1 presents only the crude OR of OASIS by SES. Model 2 additionally adjusted for age, demonstrated decreased OR, with maternal age explaining 33.3% and 47.8% of excess risk of OASIS in upper white-collar and lower white-collar workers, respectively. Model 3, included age adjustment, and additionally adjustment for smoking status, again demonstrating reduced OR, with non-smoking alone explaining 44.7% and 33.3% of excess OASIS risk in upper white-collar and lower white-collar workers. In Models 4 and 5, we additionally adjusted for birthweight and mode of delivery, with birthweight explaining 36.8% and 16.7% of excess risk, and with mode of the delivery explaining 21.1% and 0% of excess OASIS risk between SES groups. Finally, Model 6 shows that age, smoking, birthweight and mode of delivery explained up to 39.5% of differences in OASIS risk between SES groups.

**Table 4:** Odds ratios (ORs) of OASIS in nulliparous women including women admitted for first vaginal delivery after a prior cesarean section after adjustments for age and risk factors for OASIS.

	Model 1, crude	Model 2, adjusted by SES and age		Model 3, adjusted by Model 2 and smoking		Model 4, adjusted by Model 2 and birthweight		Model 5, adjusted by Model 2 and mode of delivery		Model 6, adjusted by Model 2 and smoking, birthweight and mode of delivery	
	OR (95% CI)	OR (95% CI)	Diff. with 1 (%)*	OR (95% CI)	Diff. with 2 (%)*	OR (95% CI)	Diff. with 2 (%)*	OR (95% CI)	Diff. with 2 (%)*	OR (95% CI)	Diff. with 2 (%)*
SES											
Upper white-collar	1.57 (1.39−1.78)	1.38 (1.23−1.44)	33.3	1.21 (1.07−1.38)	44.7	1.24 (1.10−1.41)	36.8	1.30 (1.15−1.47)	21.1	1.23 (1.08−1.39)	39.5
Lower white-collar	1.23 (1.12−1.35)	1.12 (1.02−1.23)	47.8	1.08 (0.98−1.18)	33.3	1.10 (1.00−1.21)	16.7	1.12 (1.02−1.24)	-	1.08 (0.98−1.19)	33.3
Blue-collar	1	1	-	1	-	1	-	1	-	1	-
Other ^a^	1.35 (1.22−1.48)	1.32 (1.20−1.46)	8.6	1.28 (1.16−1.41)	12.5	1.31 (1.19−1.44)	3.1	1.34 (1.22−1.48)	-	1.30 (1.18−1.44)	6.3
Missing	1.64 (1.48−1.82)	1.58 (1.42−1.75)	9.4	1.55 (1.39−1.72)	5.2	1.59 (1.43−1.76)	-	1.57 (1.41−1.74)	1.7	1.56 (1.40−1.73)	3.4

SES =  socioeconomic status, ^a^ The ‘other’ group included entrepreneurs, students, retired, unemployed, housewives and unclassifiable cases.

* The contribution of risk factors for OASIS to socioeconomic disparities in OASIS were measured by the percentage reduction in the odds ratio of each SES group compared to Model 2 by using formula (OR Model 2 – OR Model 3/4/5/6)/(OR Model 2 – 1).

## Discussion

For all major obstetric and perinatal complications, including preterm birth and small for gestational age [22], there is a social gradient, with women in lower socioeconomic strata being at increased risk of adverse outcomes. Generally it is believed that lower SES is associated with reduced antenatal care seeking, poorer lifestyle options and a variety of adverse environmental exposures [22], [23]. Here in contrast we identify a reverse social gradient, with nulliparous women in higher SES groups experiencing excess risk of OASIS that was in line with a single centre study from the UK [18]. Among the higher SES groups about 40% of the excess OASIS risk is mediated by age, non-smoking, higher birthweight and more interventional mode of delivery, but after full adjustment in multivariable models a large excess risk was still observed.

### Strengths and weaknesses

The most important strength of our study was that the data were derived from two large mandatory registers. In Finland, more than 99% of women give birth in publicly funded hospitals, thus our data from the Finnish MBR are almost completely population representative, with excellent validated data quality [24]. The HDR is also mandatory and its completeness and quality are high [25]. In Finland, midwives take care of all spontaneous vaginal births, limiting potential for differential misclassification of OASIS outcomes by profession of accoucheur [26].

Although a number of risks are known to be associated with SES, a limitation of the study was that we were not able to include all risk factors for OASIS found in previous studies, such as duration of the active second stage of birth [27], position of the fetal head [28], [29] and use of perineum protection techniques [30]. Although, the content of the MBR offered a unique possibility to use socioeconomic factors as exposures, the definition of parturients’ SES was challenging since they are typically young, more often enrolled as students or taking care for their children full-time. We were only able to assign SES groups based on the maternal occupation at the time of birth, since no information on the father’s occupation was available. Furthermore, education and income would have provided valuable additional information about SES, but maternal occupation at birth is related to these in Finland, and is an appropriate, available indicator for studies on socioeconomic health differences in the perinatal period [20], [31]. Information on SES was self-reported and optional and due to sensitive nature of the information it is not provided by all women. However, cases with missing SES were spread across different SES groups, since their exposures and outcomes were close to the general population, implying that any bias from missing SES data was unlikely to affect the results significantly.

SES has shown to affect pregnancy outcomes in multiple ways since lifestyle such as smoking, physical activity and nutrition, occupational such as physical demand of work and environmental factors such as neighborhood are substantially socially patterned. Smoking is one such modifiable lifestyle factor that was associated with lower OASIS incidence due to unknown reasons, but it might be suggested that smoking may interfere with collagen synthesis and connective tissue properties [32]. In this context, smoking had a beneficial effect on maternal outcomes, although in view of its major association with adverse neonatal outcomes [33], clearly maternal smoking should still be discouraged. The Finnish MBR has been shown to cover smoking habits during pregnancy relatively well [34] but a possible limitation was that smoking during pregnancy was based on self-reporting. Notably we did not observe a dose-response effect between persistent smoking and smoking discontinuation, and it might be that some women categorized as giving up smoking, actually continued smoking through pregnancy. Our data did not provide information on the number of cigarettes smoked each day and therefore, we could not further examine for a dose-response effect.

Overall, about 60% of socioeconomic disparities in OASIS incidence between SES groups remained unexplained. Herein, it might be speculated that other lifestyle, occupational and environmental factors played a role. Previous studies have demonstrated an association between smoking and bladder symptoms, [10] between low physical activity, smoking and UI [11], and between hard occupational work and UI [35]. Furthermore, a few previous studies have suggested that SES [35], [36] and low income are associated with pelvic organ prolapse [37]. We can speculate that repeated pelvic floor distension associated with physical/manual occupation increases perineal tissue flexibility and protects against perineal tears. Furthermore, connective tissue properties might vary by SES groups due to smoking, nutritional status, dietary micronutrients and physical activity not measured in the present study.

Our results confirm previous studies that suggested first vaginal delivery after a prior CS, high birthweight, vacuum and forceps deliveries [2], [5] and advanced maternal age [4] are associated with increased incidence of OASIS. Furthermore, the considerably increased OASIS incidence during the study period was in line with a trend in other Nordic countries [2], [38]. Despite the large influence of SES, we did not observe substantial changes in our estimates neither for effect sizes of the major risk factors, nor for the secular trend in OASIS incidence.

We reported the risk profile of OASIS using a large 20-year register-based dataset covering the total population in Finland. Our results demonstrate up to 18% excess OASIS incidence among nulliparous women in the highest SES groups compared with the lowest SES group. In the order of 40% of the disparity in incidence was explained by age, non-smoking, birthweight and mode of delivery. The remaining 60% of the excess risk in the highest SES groups must be explained by either unmeasured lifestyle or environmental factors, or inequalities in providing healthcare. Although reduction in OASIS incidence is widely regarded as a target for quality obstetric care provision, much evidence suggests that in particular partial OASIS remains under-diagnosed. Women who sustain unrecognised OASIS will not have appropriate primary repair, and may suffer with persistent anal incontinence secondary to unsutured sphincter defects. Paradoxically then, the positive social gradient we observe may be consistent with evidence of better obstetric care provision for women in higher social strata. Due to publicly funded health care services with free-access it seems unlike. However, these results prompt both closer examination of socially patterned inequalities in OASIS diagnosis, and further consideration of social gradients in post-partum anal incontinence.
